# Drawing a Line: Grasses and Boundaries

**DOI:** 10.3390/plants8010004

**Published:** 2018-12-25

**Authors:** Annis E Richardson, Sarah Hake

**Affiliations:** 1 Plant and Microbial Biology, University of California, Berkeley, CA 94720, USA; annisrichardson@berkeley.edu; 2 USDA Plant Gene Expression Center, 800 Buchanan Street, Albany, CA 94710, USA

**Keywords:** grass, ligule, organogenesis, boundaries

## Abstract

Delineation between distinct populations of cells is essential for organ development. Boundary formation is necessary for the maintenance of pluripotent meristematic cells in the shoot apical meristem (SAM) and differentiation of developing organs. Boundaries form between the meristem and organs, as well as between organs and within organs. Much of the research into the boundary gene regulatory network (GRN) has been carried out in the eudicot model *Arabidopsis thaliana*. This work has identified a dynamic network of hormone and gene interactions. Comparisons with other eudicot models, like tomato and pea, have shown key conserved nodes in the GRN and species-specific alterations, including the recruitment of the boundary GRN in leaf margin development. How boundaries are defined in monocots, and in particular the grass family which contains many of the world’s staple food crops, is not clear. In this study, we review knowledge of the grass boundary GRN during vegetative development. We particularly focus on the development of a grass-specific within-organ boundary, the ligule, which directly impacts leaf architecture. We also consider how genome engineering and the use of natural diversity could be leveraged to influence key agronomic traits relative to leaf and plant architecture in the future, which is guided by knowledge of boundary GRNs.

## 1. Organogenesis

Organogenesis is the self-organizing process in which complex tissues arise from pluripotent progenitors and is common to all multicellular organisms. In plants, the process of organogenesis extends beyond embryogenesis, which enables them to continually produce organs. All aerial organs arise as relatively simple-shaped primordium on the periphery of the shoot apical meristem (SAM), which contains the pluripotent stem cells. The first molecular marker of organogenesis is the downregulation of class 1 *KNOTTED-LIKE HOMEOBOX* (*KNOX)* genes in the peripheral zone of the SAM [1,2,3]. This earliest stage of primordium growth is referred to as the P0, with the plastochron stage (P) as the time between successive primordium initiations. 

The spacing of organ primordia around a SAM (the phyllotaxy) is self-organizing and highly robust. Phyllotaxy is determined by the distribution of the phytohormone auxin, which is influenced by the directional export of auxin by the PIN-FORMED transporters (PIN). This process is a self-organizing feedback loop, and the spacing between each primordium is predicted to be influenced by the size of the region of auxin depletion around the older primordium [4,5,6,7,8,9,10]. The formation of PIN1 convergence points in the SAM of the model eudicot plant *Arabidopsis thaliana* is essential for organ initiation [11,12,13,14,15]. This PIN1 convergence point leads to the formation of an auxin maximum and the subsequent downregulation of *KNOX* genes, which allows differentiation and outgrowth of organ primordia.

## 2. Boundaries and Plant Development

A fundamental step in organogenesis of multicellular organisms is the delineation of distinct populations of cells by forming boundaries. Boundary formation is essential for the function of the mature organ since it allows correct patterning and the segregation of different activities. In the case of vegetative development in plants, the formation of a boundary between the SAM and the incipient primordia is essential for both maintenance of the stem cell population and the correct shape of the mature organ [16]. This meristem/organ boundary allows for the separation of the cells that will become determinate and form the organ, while those that retain an indeterminate state maintain the meristem. 

Meristem/organ boundaries are characterized by low division and expansion rates, parallel oriented microtubules, and relatively stiff cell walls [17]. These features contrast with the high cell division and cell expansion rates, low cell wall stiffness, and perpendicular oriented microtubules in the primordium tissue. The difference between the tissue properties of boundaries and the primordium generates conflict within the tissue, which allows the physical bulging of the primordium from the surface of the meristem [18,19,20]. The distribution of differentially growing regions can then generate distinct shapes [21]. Therefore, in addition to roles in separation of functionally different tissues, boundaries also contribute to organ shape through differential growth patterning [22]. 

Boundaries also form within the organ itself, delineating different tissues. These within-organ boundaries can have central roles in the final organ shape. For example, the juxtaposition of the abaxial and adaxial tissues in the leaf are essential for lamina outgrowth [23,24,25]. Within-organ boundaries can also be elaborated, contributing to morphological diversity. For example, stipules form at the base of the petiole in eudicot leaves such as peas [26]. Boundary regions can also be elaborated in mutants in response to ectopic gene expression. For example, ectopic *KNOTTED1* expression in the lemma/awn boundary in the barley *Hooded* mutant, results in the formation of a “hood” structure consisting of an ectopic floral meristem and triangular lateral outgrowths [27,28,29,30,31]. Similarly, ectopic *KNAT1* expression leads to meristems forming in the boundary regions of the lobed leaf [32].

### 2.1. The Boundary Gene Regulatory Network 

**Figure 1:**
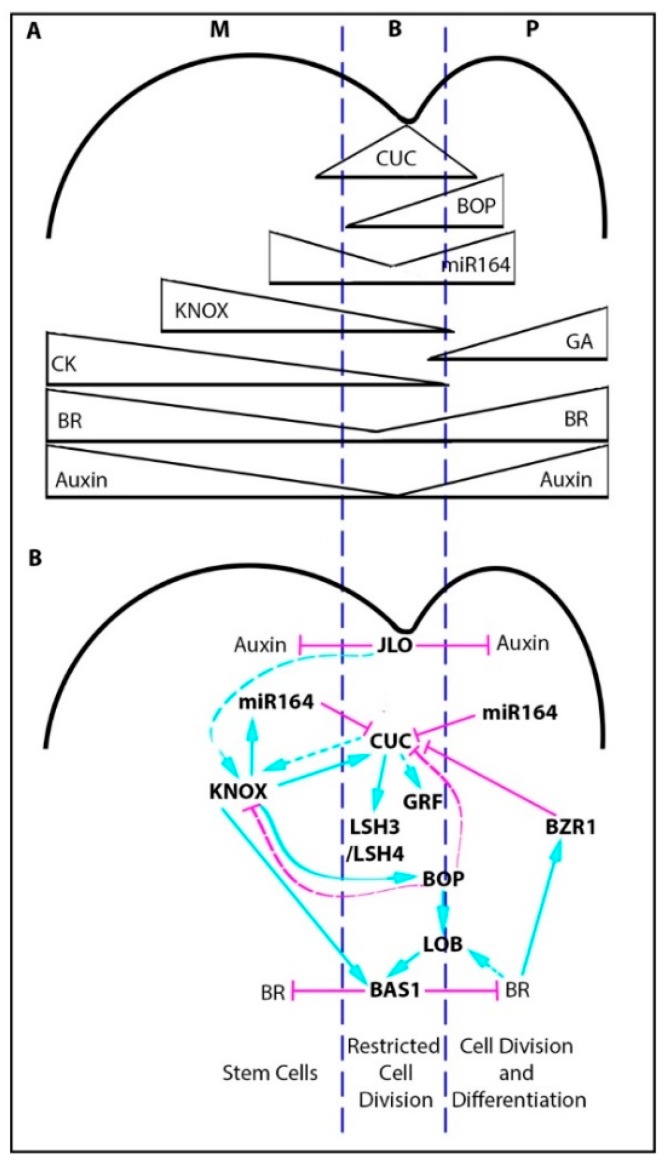
**Regulation of meristem-organ boundaries in *Arabidopsis*.** (**A**) Gradients of a selection of gene expression patterns and hormones across the meristem/organ boundary. (**B**) A summary of the gene regulatory network involved in meristem/organ boundary specification. Blue arrows indicate positive regulation while magenta lines indicate negative regulation. Solid lines represent direct regulation, dashed lines, indirect regulation. The dark blue lines delineate the meristem (M), boundary (B), and primordium (P) regions.

Most of our understanding in how meristem/organ boundaries are defined has come from genetic studies in *Arabidopsis*. Of particular importance are mutants that have a fused organ phenotype, including *cup-shaped-cotyledon1/2/3 (cuc), growth regulating factor (grf)*, and *lateral organ boundary (lob)* mutants [16,33,34,35,36], which highlighted key boundary genes. This body of work has shown that boundary specification requires a complex network of transcription factors, miRNAs, and hormone interactions summarized in Figure 1. Central players include the NAC domain transcription factors, *NO APICAL MERISTEM* (*NAM*, or *AtCUC1,* and *AtCUC3*), which are regulated by miR164, and are part of a feedback network with the *KNOX* gene, *SHOOTMERISTEMLESS* (*STM*) [33,34,35,37,38,39,40,41,42,43,44,45,46]. The CUC transcription factors also directly regulate the expression of other boundary genes, such as *LIGHT-DEPENDENT SHORT HYPOCOTYLS 3* and *4* (*LSH3* and *4*), which are proposed to suppress organ differentiation [37]. Downstream of the *CUC* genes, *GRFs* are also expressed in the boundary, which play a role in the suppression of cell division and expansion [34].

Low growth rates in the boundary are also influenced by the spatial distribution of growth promoting hormones like auxin and brassinosteroids (BR) [47]. Both auxin and BR maintain higher levels in the meristem and developing primordia, and low levels in the boundary. Low auxin levels in the boundary are influenced by JAGGED LATERAL ORGANS (JLO) [39]. High BR in the primordium feeds back to regulate the spatial expression of the *CUC* genes, which limits them to the boundary domain. This inhibition is through BR promotion of *BRASSINAZOLE-RESISTANT 1* (*BZR1*) expression, which inhibits *CUC* expression. Low BR levels in the boundary are influenced by the expression of *PHYB ACTIVATION TAGGED SUPPRESSOR1* (*BAS1*), which is a BR inactivating enzyme [35]. The expression of *BAS1* is regulated directly by the boundary gene *LOB1*, and BR can influence *LOB* expression forming a reinforcing feedback loop [35], restricting low BR to the boundary domain. 

The boundary region is further refined through the activity of the ankyrin repeat proteins BLADE-ON-PETIOLE 1 and 2 (BOP1 and 2), which are localized to the base of the developing primordium. BOP activity results in the repression of *CUC* gene expression in the base of the primordium, and promotes the expression of *LOB* transcription factors [48,49].

Overall, these feedback networks maintain a clear boundary domain delineating the two distinct populations of cells (the meristem and the differentiating primordium) and spatially pattern distinct growth patterns.

### 2.2. The Boundary Gene Regulatory Network and Leaf Margin Development

The boundary gene regulatory network also has a role in the elaboration of leaf margin development, in particular, influencing serration and compound leaf development in eudicot systems including *Arabidopsis*, *Cardamine hirsuta*, tomato, and pea [50,51,52,53,54,55]. 

Despite the fact that many of the boundary components are shared between species, work in diverse eudicots has highlighted key differences in the network when it has been co-opted for margin development. For example, in tomato the *KNOX* gene *TKn1* is sufficient to initiate compound leaf formation. However, in peas *KNOX* genes do not have a role in compound leaf development and the pea ortholog of LEAFY (UNI) is sufficient to initiate compound leaf development [56,57]. LFY/UNI in peas allow formation of compound leaves by promoting indeterminacy in the margin, while LFY in inflorescences cause determinate growth in flowers. In tomatoes, gibberellic acid (GA) inhibits leaf complexity but, in peas, GA promotes leaf complexity [50]. This co-option of the boundary network in margin elaboration and the variations between eudicot species illustrates that different plant species can use the same regulators to induce opposite effects. Different eudicots also use specific factors to modulate leaf margin development. For example, the homeodomain protein RCO functions to inhibit growth in the boundary of developing leaflets in *Cardamine hirsuta*. *RCO* is specific to the core *Brassicacae* but was lost in *Arabidopsis* [58].

Given the profound effect that boundary specification can have on leaf shape and plant productivity, translating this research into crop species is vital. This translation is especially important when considering future aims of developing accurate computational models of crop growth and development to help predict the effects of a changing climate on crop productivity. In this paper, we review the current understanding of boundary specification during vegetative development, and the effects on leaf morphology in grass crops in comparison with eudicot models (Table 1). 

## 3. Vegetative Organogenesis in Grass Crops 

**Figure 2:**
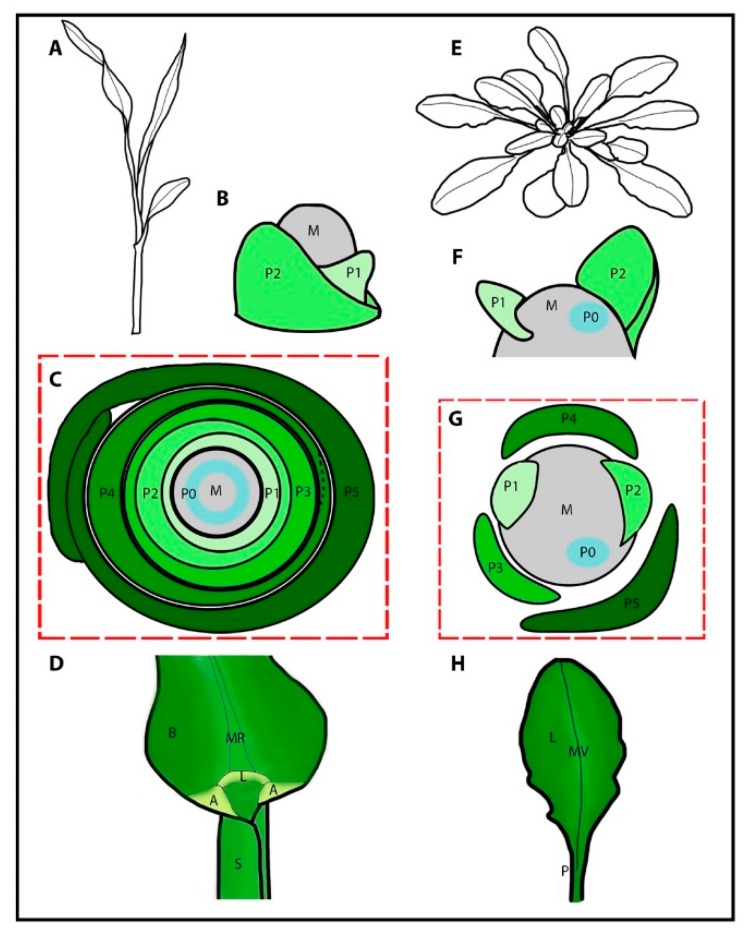
**Grasses have distinct leaf and primordium shapes.** A comparison of the morphology of the grasses versus *Arabidopsis* during vegetative development. (**A**) A cartoon of a grass seedling. (**B**) A cartoon representation of a grass vegetative meristem (M) with the first and second leaf primordia shown (P1 and P2) encircling the meristem. (**C**) A cartoon of a transverse cross section through a grass seedling, showing how each successive leaf (P1–P5) encircles the meristem and the younger leaves. The P0 is the region of *KNOX* gene expression down-regulation, which forms a ring. The sheath margin boundaries are not defined until P4 (dotted line) after which the margins are separate (P5). (**D**) A cartoon of the blade/sheath boundary in a mature grass leaf, depicting the blade (B), midrib (MR), ligule (L), auricles (A), and the sheath (S). (**E**) A cartoon of an *Arabidopsis* plant during vegetative growth. (**F**) A cartoon representation of an *Arabidopsis* vegetative meristem (M) with the first and second leaf primordia shown (P1 and P2), which do not encircle the meristem. (**G**) A cartoon of a transverse cross section through an *Arabidopsis* seedling, showing each successive leaf (P1-P5). (**H**) A cartoon of a mature *Arabidopsis* leaf, depicting the lamina (L), midvein (MV), and the petiole (P).

Most of the major food crops, including wheat, rice and maize, are members of the grass family (*Poaceae*) and are part of the monophyletic clade called the monocots. The monocots diverged from eudicot species 150 mya [59]. Monocots have distinct leaf shapes, generally sharing an ensheathing leaf base and parallel venation. These shape differences between monocots and eudicots are clear from the earliest stages of organogenesis (Figure 2A–H).

Unlike eudicot models in which the P0 is a point on the SAM that grows out to form a peg-like outgrowth (Figure 2F–G), in the grasses, the leaf P0 encircles the SAM (Figure 2B–C), and is referred to as the disc of insertion [60,61]. This disc of insertion forms the ensheathing leaf base. Each successive leaf base encircles both the meristem and all younger leaves, forming whorls containing a single leaf (Figure 2C). Like eudicot models, auxin accumulation followed by the downregulation of class 1 *KNOX* genes [1] is central to organ initiation in the grasses (Figure 2C). When auxin signaling is disrupted, as is the case when maize SAMs are treated with the auxin inhibitor NPA (N-1-naphthylphthalamic acid), organ initiation and *KNOX* downregulation is halted [62]. Auxin signaling is, therefore, central to recruitment of cells into the primordia in grasses, which is similar to eudicots.

Auxin maxima are formed by convergence points of PIN1 in *Arabidopsis* meristems. In contrast to *Arabidopsis*, the grass model, *Brachypodium distachyon*, has two *PIN1* orthologues known as *PIN1a* and *PIN1b,* and a sister clade to *PIN1*, *SISTER OF PIN1* (*SoPIN1*), with each showing sub-functionalization. This is independent of transcriptional control. PIN1a and PIN1b accumulate in the vasculature and the *pin1a/pin1b* double mutant has short internodes. SoPIN1 forms convergence points in the inflorescence meristem and the mutant has organ initiation defects similar to the *Arabidopsis pin1* mutant [63,64]. The *SoPIN1* clade is not unique to the grasses and is found in eudicots, including *Medicago truncatula* and tomato, but was lost in the *Brassicaceae* family. Mutants in the *SoPIN1* clade in *Medicago* and tomato (*entire2*) show pleiotropic effects including defects in leaf development [65,66], which suggests that *SoPIN1* could have a role in grass leaf development even though it was not reported for *Brachypodium* [63].

Once the disc of insertion has been specified, the ring-shaped P1 primordium (Figure 2B), goes on to develop into a grass leaf with a modular structure (Figure 2A). The wrapped lower leaf region known as the sheath provides structural support. The middle hinge region regulates a leaf angle and develops two distinct structures including the ligule, which is a fringe of tissue proposed to act as a sliding gasket, and two triangular auricle regions at the leaf margin, which influence the leaf angle (Figure 2D). The upper region known as the blade bends away from the plant and intercepts light. The interaction between these three regions influences plant height and the leaf angle, which has significant impacts on plant productivity [67,68,69,70,71,72,73,74,75]. These traits are of high agronomic importance since they can directly affect the yield of an individual plant, and the yield of an entire field when planting density is taken into consideration.

Clonal sector analyses of grass leaf development have shown that during the earliest stages of leaf primordium development from P0 to P3, only the blade forms. Then from P3–P4, the sheath margins arise from an overlapping region in the disc of insertion [61,76] and the ligule/auricle region begins to differentiate [77]. Therefore, during the earliest stages of grass leaf development, three different boundaries need to be specified for correct leaf shape; the ring-shaped meristem/organ boundaries (which go on to become the boundaries between the leaf whorls), the intra-whorl boundaries (the boundary between the sheath margins) and a within-organ boundary (the boundary between the sheath and blade where the ligule and auricle form) (Figure 2C–D). 

## 4. Boundary Specification in Grass Crops

### 4.1. Meristem/Organ Boundaries in the Grasses

Meristem/organ boundaries in the grasses form an encircling ring (Figure 2B,C). Without correct specification of this boundary, delineation between the stem cells (the SAM) and the differentiating primordium cells fails to occur. A lack of separation between differentiating and pluripotent cells results in the termination of the meristem, as observed in mutants such as the *cupuliformis* in *Antirrhinum*, *cuc1/2/3* in *Arabidopsis*, and *nam* in Petunia, which all have mutations in the *NAC* domain transcription factor family *NAM/CUC3* [16,36,78,79]. These classic meristem-organ boundary mutants also develop fused leaves and floral organs due to the lack of *KNOX* gene downregulation in the organ boundaries. Thus, highlighting the role of boundaries in maintaining the separation between successive whorls of organs as well as maintaining the meristem. Conversely, meristem-like activity can spread into the leaf if the boundary is not maintained. In the case of the *blade-on-petiole* (*bop1*) mutant in *Arabidopsis*, *KNOX* gene activity, which is indicative of a meristem-like identity, spreads into the leaf base resulting in the formation of ectopic leaf tissue [80].

Grass genomes have representatives of the core genetic elements in the *Arabidopsis* meristem-organ boundary regulatory network (Table 2). For example, the *NAM* and *CUC3* genes, and the core miRNA164-NAM module likely predates the monocot/eudicot split [81,82,83]. Rice has one representative of NAM (Os06g0267500) and CUC3 (Os08g0511200), but maize, like *Arabidopsis*, has two NAM genes (GRMZM2G139700 and GRMZM2G393433) and one CUC3 (GRMZM2G430522), which illustrates gene duplication of the NAM family outside of the Brassicas [51]. The expression pattern of Zm*CUC3* in maize lateral organs and the SAM mirrors that in eudicots, although the patterns of Zm*CUC3* and the Zm*NAM*1/2 genes during embryo development differ [81]. Similarly, maize has a recent duplication of the *AtBOP1/2* genes called *TASSELS REPLACE UPPER EARS 1* (*ZmTRU1*, GRMZM2G039867) and *TRU1-like* (*ZmTRL1*, GRMZM2G060723), whereas rice has only a single gene, *OsBOP* (Os01g72020) [84].

Unlike the *NAM/CUC3* and *BOP* genes, some of the gene families implicated in meristem-organ boundary specification are enlarged in the grasses. For example, where there are three members of the miR164 family in *Arabidopsis* (miR164a,b,c), there are six reported in rice (miR164a,b,c,d,e,f) and eight in maize (miR164a,b,c,d,e,f,g,h) [85]. In *Arabidopsis*, the three miR164 family members are functionally redundant but exhibit expression domain differences suggesting some sub-functionalization [86]. miR164b has a role in regulating NAC transcription factor expression during lateral root formation in maize, which indicates a function in patterning lateral outgrowths [87]. The roles of other miRNA164 family members in grasses are yet to be elucidated, especially considering the large size of the NAC transcription family (for example, rice has 149 members) [88]. This expansion of gene families may provide the opportunity for sub-functionalization of key boundary regulatory genes in the grasses.

Forward genetic screens in grasses have identified mutants with tube and fused leaves, which could be indicative of mutations in meristem/organ boundary regulation genes. So far, these mutants with fused leaf phenotypes such as rice *onion-1, 2,* and *3*, maize *adherant1*, and *fused leaves1* (*fdl1*), have defects in epidermal wax deposition and are not associated with any of the canonical boundary regulatory genes, such as *NAM* or *CUC3* genes [89,90,91,92,93,94]. The lack of *nam/cuc3* family mutants could suggest functional redundancy in the grass family, or that the mutations are embryo lethal, which implies that the leaf phenotype cannot be observed. 

Although no *nam* or *cuc3* mutants have been reported in the grasses, mutants in the orthologues of several boundary genes are known. Two orthologues of *AtBOP1* are found in maize known as *TRU1* and *TRL1*. The maize *tru1* mutant does not have a leaf phenotype, although the protein accumulates in an interesting sheath pattern [84]. *ZmTRL1* has no reported mutant phenotype. The two genes may be partially redundant with respect to vegetative organ boundary specification. In barley, the *AtBOP1* orthologue *HvCUL4*, has a defect in leaf development, with the *cul4* mutant showing a displacement of ligule/ auricle tissue [95]. This may mirror the displacement of distal identities within the proximal tissue observed in *Arabidopsis*. 

Arabidopsis *lob* mutants have fusions between cauline leaves and branches but normal vegetative organs. *LOB* is expressed at the base of lateral organs and plays a role in negatively regulating BR signaling in boundaries [33,35]. Double and triple mutant analysis of the homologues of *AtLOB* show no additional phenotypes, but expression analysis highlights distinct expression patterns, suggesting sub-functionalization based on changes in the expression pattern rather than in a coding sequence [96]. In maize, the function of one homolog of *AtLOB* has been examined so far, *RAMOSA2* (*RA2*). RA2 regulates axillary meristem formation during inflorescence development. However, there are no reported organ fusion phenotypes in the *ramosa2* mutant, which contrasts with the AtLOB function [97].

The apparent conservation of boundary gene families suggests a common mechanism for meristem/organ boundary specification in eudicots and grasses, but the exact roles of the genes in grasses are yet to be understood. Some examples studied so far, such as *RA2,* illustrate diversity in gene function.

### 4.2. Intra-whorl Boundaries (the Boundary Between the Overlapping Margins of the Sheath) in the Grasses

The sheath arises from an overlapping region in the disc of insertion during early P3/ late P4 development, requiring the formation of a new boundary between the two sheath margins (intra-whorl boundary) (Figure 2C, P4 dotted line). The delineation is shown clearly by the expression of adaxial and abaxial markers in the region of the incipient sheath margins [98]. Separation of sheath margins is dependent on auxin since the sheath remains fused and tube-like when plants are cultured in the presence of the auxin inhibitor NPA. In support of this dependency, expression of auxin biosynthesis genes such as *SPARSE INFLORESCENCE 1* (*SPI1*, a *YUCCA* gene) is observed at the incipient sheath boundary. *ZmNAM2* (also called *ZmCUC2*) is also expressed in this region, which suggests the recapitulation of the meristem-organ boundary specification at this location and stage in grass leaf development [98]. 

What specifies or activates this intra-whorl boundary pathway forming the sheath margins is not clear. The *narrowsheath1*/2 double mutant in maize lacks this region, suggesting a role for NS1/2 in patterning or growth of this region [99]. Comparisons of monocots with fused sheaths, such as seen in some members of the sedges, could help elucidate this component in grass sheath development, highlighting factors involved in the evolution of the grass leaf.

### 4.3. Within-Organ Boundaries (the Blade/Sheath Boundary and the Development of the Ligule and Auricle) in the Grasses

The boundary between the grass leaf sheath and the blade develops characteristic structures; the ligule and the auricle; which directly influence the leaf angle, and can be used to define different species. 

The first indication of the ligule during maize leaf development is an apparent increase in cell divisions in both a transverse and longitudinal direction in the adaxial epidermis to form the pre-ligule band [77]. Shortly thereafter, a reoriented accumulation of ZmPIN1a in the epidermis is observed, suggesting that, like organ initiation in the meristem periphery, auxin signaling is important in ligule formation and outgrowth [100]. Laser capture RNAseq of developing ligules found that ligule development involves the recapitulation of the meristem/organ boundary network within the developing leaf [101], highlighting roles for transcription factors such as *ZmNAM2* in addition to auxin, giberellic acid (GA), cytokinin (CK), and brassinosteroid (BR) signaling. This RNAseq dataset suggests that, like eudicot leaf margin modification, the grasses have recruited a common boundary specification network in the development of a novel leaf morphology. 

Mirroring the diversification observed in leaf margin development in eudicots, analysis of grass mutants with defects in the ligule/auricle boundary have identified species-specific components. The many blade/sheath boundary mutants in maize, barley, and rice highlight the role of different genes. Some appear to be specific to the outgrowth of the ligule, while others influence the specification of the blade/sheath boundary. 

#### 4.3.1. *Liguleless* Mutants and the Patterning of the Ligule

**Figure 3:**
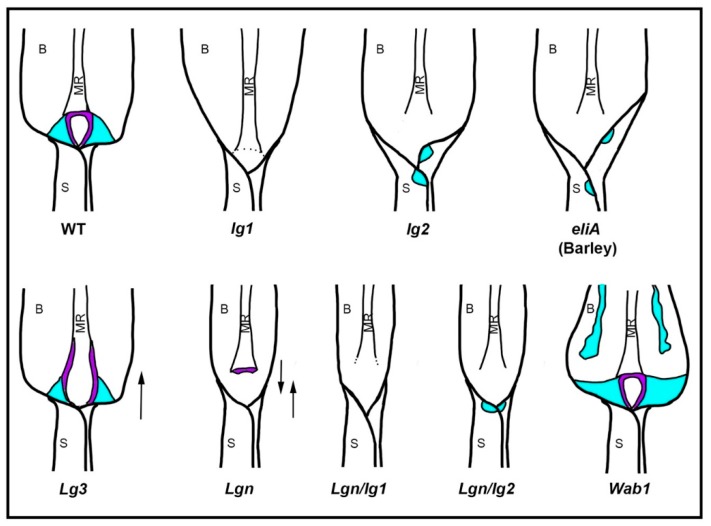
**The morphology of the leaf blade/sheath boundary in reported ligule mutants.** Cartoons depicting the typical morphology of the blade/sheath boundary in mature leaves of wild-type (WT), *liguleless1* (*lg1*), *liguleless2* (*lg2*), *eligulumA* (*eliA*, a barley mutant), *Liguleless3* (*Lg3*), *Liguleless narrow* (*Lgn*), double *Lgn/lg1*, double *Lgn/lg2*, and *Wavy Auricles in Blade* (*Wab1*) plants. In each cartoon, the blade (B), midrib (MR), and sheath (S) are labelled. The ligule (purple) and the auricles (cyan) are also highlighted. Where the mutant leaf lacks a ligule, but retains a clear boundary between the blade and sheath, the boundary is indicated by a dotted line. The arrows indicate the direction of the displacement of the sheath tissue in the mutant.

LIGULELESS1 and 2 (LG1 and LG2) are grass-specific transcription factors belonging to the squamosa binding transcription factor and BZIP/DOG domain transcription factor families, respectively. In maize, *lg1* mutants retain a clear blade sheath boundary, but lack the ligule and auricle [102] (Figure 3). In rice and barley, *lg1* mutants are more severe than in maize, completely lacking the ligule region in all leaves [103,104]. The milder phenotype of maize may be explained by duplicates of *ZmLG1*. *LG1* is expressed in the pre-ligular band [101] and acts cell autonomously, which suggests that LG1 functions to specify the ligule [105]. *lg2* mutants, in contrast, have a diffuse blade/sheath boundary and retain reduced auricles at the margins which are displaced vertically relative to each other (Figure 3). *lg2* mutant phenotypes are yet to be described in other grasses. *ZmLG2* has a broad expression pattern but a specific protein localization, and it is able to act non-cell autonomously. The phenotype of *lg2* has led to the hypothesis that LG2 may have a role in defining the blade/sheath boundary itself [105,106]. Double mutant analysis in maize has suggested that both LG1 and LG2 act in the same pathway [105], with *LG2* being expressed earlier than *LG1* [100,106,107].

RNAseq of *lg1* mutants showed an enrichment of differentially expressed genes involved in auxin signaling, in addition to MYB and SBP transcription factors [101]. The directly bound and modulated targets of LG1 and LG2, however, are yet to be identified. Given data from other species, LG1 and LG2 may form heterodimers with other transcription factors. For example, in *Arabidopsis*, the BZIP DOG domain transcription factor PERIANTHIA (a member of the same clade of BZIP transcription factors as LG2 [108]) is involved in floral development, and interacts with BOP1 and 2 in yeast [49]. The barley orthologue of *AtBOP2*, *UNICULME4* (*HvCUL4)* functions in axillary meristem development and in ligule specification [95]. These observations lead to the hypothesis that LG2 may interact with BOP homologues in the grasses to pattern the blade/sheath boundary.

In addition to homo-dimerization and hetero-dimerization, BZIP transcription factor activity has been shown to be post-translationally regulated via phosphorylation [109]. LIGULELESS NARROW (LGN) is a serine-threonine kinase that is non-functional in the dominant mutant, *Lgn-R*. *Lgn-R* mutants have a pleiotropic phenotype including narrower leaves, the loss of the ligule except at the midrib, and a diffuse blade/sheath boundary (Figure 3). This mutant has led to the hypothesis that a phosphorylation cascade propagates the ligule signal from the midrib to the margins of the leaf. A role for phosphorylation was also highlighted by network analysis where the authors proposed that a membrane associated kinase regulator (MPKR) could act with bHLH transcription factors to influence brassinosteroid (BR) signaling in the ligule [110]. Mutants in rice with reduced BR synthesis such as *dwarf4*-1, *ebisu dwarf* (*d2*), *brassinosteroid*-*deficient dwarf 1* (*brd1*), or BR signaling, such as *d61*, have more upright leaves [111,112,113,114]. Similarly, RNAi knock-down of the BR signaling components, *OsBAK1* in rice and *ZmBRI1* in maize, have reduced BR signaling and more upright leaves with reduced auricles [115,116]. The maize *brd1* mutant has reduced BR synthesis with defects in ligule and auricle development [117]. *ZmBRD1* is expressed in the base of P3 leaves [101], which overlaps with the localization of TRU1 [84]. These results suggest that phosphorylation cascades and BR may be involved in mediolateral patterning of the blade/sheath boundary. 

The barley liguleless mutant *eligulumA* has a diffuse blade/sheath boundary (Figure 3) and carries a mutation in a gene that encodes a protein with an RNaseH domain but otherwise of unknown function [118]. In barley, *ELIA* is expressed in an overlapping domain with *LG1*. Although no *eligulum* mutant has yet been reported in maize, gene network analyses highlight a module expressed in the pre-ligule band that includes both maize homologues of ELIA [110]. These results suggest that ELIA may play an, as yet, unknown role in the blade/sheath boundary specification and ligule development across the grasses.

#### 4.3.2. Ectopic Induction of New Blade/Sheath Boundaries

Several dominant maize mutants exhibit ectopic formation of new blade/sheath boundaries, suggesting an additional regulatory network involved in initiating blade/sheath boundary patterning. In support of this, the genes able to trigger ectopic blade/sheath boundaries form a distinct module from the pre-ligule patterning genes (those genes outlined in Section 4.3.1) in gene network analyses [110]. Genes able to ectopically induce new blade/sheath boundaries include the homeobox genes *KNOTTED1* (KN1), *GNARLEY1* (*KNOX4*), *LIGULELESS 3*, *LIGULELESS 4*, and the TCP transcription factor *WAVY AURICLES IN BLADE1* (*Wab1*) [2,107,119,120,121,122,123,124,125,126]. An additional ectopic blade/sheath boundary mutant, *Hairy sheath frayed* (*Hsf*), has also been identified. *Hsf* develops sheath-like prongs on the blade of the leaf [127,128] and is involved in cytokinin (CK) signaling [Michael Muszynski, Personal Communication]. These mutants suggest that KNOXs, TCPs, and CK signaling could be involved in proximal patterning of the grass leaf before ligule and auricle outgrowth occurs. 

In support of the hypothesis that *KNOX* genes are involved in this proximal/distal patterning, KNOX protein accumulates at the base of developing grass leaves, suggesting that KNOXs could provide a “proximal” patterning signal. *KNOX* expression in this boundary may provide competency to respond to the ligule and auricle patterning factors. Interestingly, the KNOX interacting factors, BEL12 and 14, are expressed in the developing ligule [101,129] and are bound and modulated by KN1 [44]. LG3, which is also expressed at the ligule, interacts with both BEL12 and 14 [Aromdee and Hake, unpublished data]. Ectopic expression of *KNOX* genes in other systems also triggers morphological changes and outgrowths. For example, ectopic expression of the *KNOX* gene *BKN3* in the barley lemma/awn boundary triggers the formation of an ectopic floral meristem and triangular marginal outgrowths. This dramatic morphological change correlates with an induced re-orientation of tissue cell polarity (as shown by the localization of SoPIN1) and the ectopic expression of boundary genes such as *NAM* [31], lending further support to the hypothesis that *KNOX* genes are able to pattern new boundary regions and morphological changes.

*WAB1* is normally expressed in developing inflorescences and is required for branch initiation in the tassel [121]. In the dominant gain of function mutant, *WAB1* is ectopically expressed in the leaf blade and induces the ectopic expression of *LG1*, which leads to auricle-like outgrowths in the blade (Figure 3). Although WAB1 does not play a role in normal leaf development, it could indicate a possible role for other TCP transcription factors in the regulation of LG1 expression in the leaf. 

The recessive mutant *extended auricles 1* (*eta1*) develops ectopic auricle tissue, and has a diffuse blade/ sheath boundary. The causal mutation of *eta1* has not been identified, but it has been shown to be involved in the same pathway as LG1 and LG2 [130,131]. ETA1 is proposed to be a possible component of the bridge between the blade/sheath boundary patterning network and the pre-ligule patterning network.

#### 4.3.3. A Proposed Model of Blade/Sheath Boundary Specification

Given that liguleless mutants maintain a blade and a sheath, it is likely that the blade/sheath boundary specification can be separated into two distinct phases. 

First, a broad domain boundary between the sheath and blade is specified early in the leaf primordium. Since there are no reported mutants which are only sheath, only blade, or a hybrid of the two identities, it is likely that this stage is genetically redundant. This phase involves factors such as *KNOX* genes, and genes associated with the sheath such as *BOP*, as well as phytohormone gradients such as auxin and cytokinin. Although *KNOX* gene expression is excluded from developing leaf primordia, the accumulation of KNOX protein in the base of the developing leaf could promote the expression of *BOP* genes, specifying the sheath domain. This would predict that the loss of function of multiple *BOP* genes with overlapping functions in the grasses would result in a loss of sheath identity, and that ectopic *KNOX* expression would induce *BOP* expression. Similarly, overexpression of a BOP gene in the developing grass leaf would increase the proportion of sheath to blade. Based on the RNAseq work by Johnston et al. and the mutant phenotypes of *Hsf*, converging gradients of auxin (distal signal) and cytokinin (proximal signal) could contribute to patterning the boundary between the sheath and blade. Early studies that added auxin transport inhibitors to maize seedlings showed a disruption of the blade sheath boundary [132]. It would be of great interest to explore the distribution of auxin and cytokinin in the developing leaf primordium using reporters, as well as to test the effects of differential hormone treatments on the ratio of sheath to blade. 

**Figure 4:**
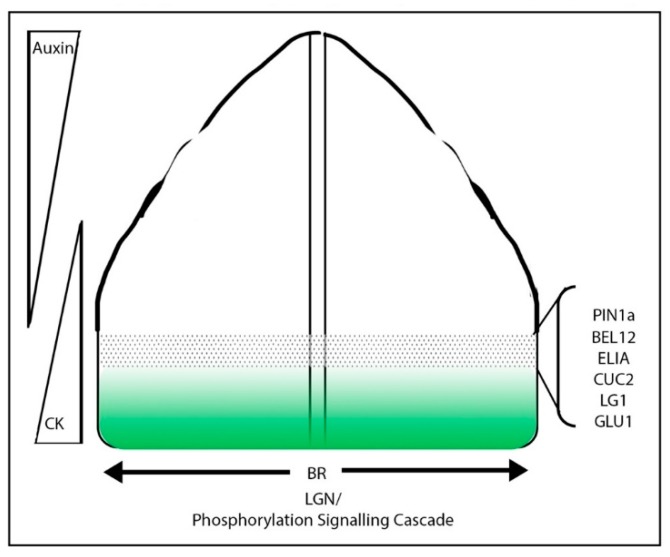
**How is a ligule patterned?** Summary of the known gene expression patterns in the developing grass leaf at P6 and the hypothetical gradients of phytohormone signaling. Green represents the expression pattern of the *BOP* homologues, which overlaps with *LG3, BRD1*, and *BEL14* in the base of the leaf. The dotted region represents the pre-ligule band, where *PIN1a, BEL12, ELIA, CUC2, LG1*, and *GLU1* are expressed. Hypothesized gradients are also illustrated for Auxin, CK, and BR.

The second phase of boundary development involves the refinement of the blade sheath boundary and the ultimate specification of the pre-ligule band at P6 (Figure 4). This phase likely involves genes expressed at the ligule and those that have liguleless phenotypes. Within this stage, we can predict factors involved in refining the boundary, and those important for ligule specification and outgrowth to function. The *lg2* mutant has a diffuse boundary, which suggests that it is involved in refining the boundary region. *lg1* has a distinct blade/sheath boundary, and is therefore likely specific to the specification and outgrowth of the ligule. The displacement of the ligule and the blade/sheath boundary in the *Lgn* mutant suggests that a phosphorylation cascade and BR signaling may be involved in propagation of the “ligule signal” out from the midrib to the margins of the leaf. It will be of great interest to look at the relative timing of ligule specific gene expression alongside PIN orientations to determine how the ligule region is defined.

## 5. Pleiotropy and Boundaries

Given the profound effects on morphology, manipulation of genes involved in boundary specification could lead to modulation of the leaf phenotype, providing a rich resource for phenotypic plasticity to be tested in different environmental and field conditions. For example, in dense planting fields, more upright leaves, especially in the upper canopy are important, whereas more sparse, inter-cropped fields, may benefit from a wider leaf angle. Many of the existing mutants, however, have pleiotropic effects, which can negatively impact yield. For example, both the maize *lg1* and *lg2* mutants have upright leaves, but also have severely reduced tassel branch numbers [102,133,134]. Similarly, the barley mutant *eliA* is pleiotropic with a shorter stature, ligule defects, and compact inflorescence spikes [118]. This pleiotropy is not unique to the grasses. For example, the *cuc2*, *cuc3*, and *lof1/2* mutants in *Arabidopsis* all have defects in branching [16,135,136,137]. The combined effects on both leaf architecture, branching and inflorescence architecture of many of these mutants, often leads to a reduced yield.

To explore whether individual phenotypic components could modulate yield, the pleiotropy needs to be broken. Pleiotropic effects could be modulated through: (1) changes in cis-regulatory elements that influence the timing or spatial distribution of expression, (2) altering tissue specific partners, and (3) modulating different tissue-specific downstream elements. For example, DELLA mutants have pleiotropic defects, affecting both stem growth and meristem size. DELLA’s effect on stem growth has been linked to direct regulation of the cell cycle inhibitor KRP2 and is independent of meristem size regulation. The genetic uncoupling of stem elongation and meristem size via modulation of KRP2 was effective in both *Arabidopsis* and barley, generating semi-dwarf plants [138]. 

In model eudicots like *Arabidopsis*, transgenics are used to overcome pleiotropic effects of key regulatory genes. The extensive transgenic toolkits in *Arabidopsis* enable cell-type specific and inducible expression systems [139] to modulate gene expression in a precise manner. For example, conditional dsRNAi silencing of CLV3 allowed identification of the specific function of CLV3 in the meristem, which uncoupled the effects from the severe global changes caused in the full *clv3* mutant [140]. In transformation tractable species, the use of genome editing via CRISPR/cas9, for example, can also be used to alter cis-regulatory elements to uncouple phenotypes. This technique has already been used successfully in tomato to combine alleles that were selected during domestication and more recent breeding for distinct flower morphology and fruit retention traits. Combining the two traits highlighted a negative epistatic relationship, which could be overcome by varying the dosage of the relevant genes using homo/heterozygote mutants, and through CRISPR/cas9 to introduce allelic variation [141]. The use of genome editing to introduce allelic variation in cis-regulatory sequences can also lead to an increase in phenotypic variation [142], which could be used as a resource to break pleiotropy. 

Transgenic approaches can be more difficult in grass crops due to the expense and time of transgenics, difficulty in transgenerational maintenance of the transgene, and public opinions regarding genetic modification. Alternatively, rich natural diversity in species such as maize, can be taken advantage of to break links in pleotropic defects. 

## 6. Conclusions 

A common underlying mechanism for boundary specification exists between eudicots and grasses, specifying meristem/organ and intra-whorl boundaries during vegetative development. In both eudicots and grass crops, this mechanism has been co-opted to specify within organ boundaries to generate morphological diversity. In both cases, however, there are species and family-specific elements that modulate the core boundary network and highlight the importance of studying boundary specification in both eudicot models and grass crops. The dynamic regulation of these boundary regulatory networks could yield rich phenotypic diversity in agronomically important traits such as leaf angle, making use of targeted natural variation or genome editing in key nodes of the network.

## Figures and Tables

**Table 1:** Glossary of studied related genes in *Arabidopsis*, maize, and barley mentioned in the review.

*Arabidopsis*	Maize	Barley	Description
SHOOTMERISTEMLESS (STM)	KNOTTED1 (KN1)	BARLEY KNOTTED 3 (BKn3)	KNOX transcription factor
CUP-SHAPED COTYLEDON 1,2,3 (CUC1, 2, 3)	NO APICAL MERISTEM 1 and 2 (NAM 1,2), CUC3		NAC domain transcription factor
BLADE ON PETIOLE 1 (BOP)	TASSELS REPLACE UPPER EARS 1 (TRU1) and TRU1-like	UNICULME4 (CUL4)	Ankyrin repeat domain protein
miR164 a/b/c	miR164 a/b/c/d/e/f/g/h		microRNA
PIN-FORMED 1 (PIN1)	PINFORMED 1a and 1b (PIN1a, PIN1b)		Auxin transporter
not present in *Arabidopsis*	SISTER OF PIN1 (SoPIN1)		Auxin transporter

**Table 2:** Glossary of grass gene names. Where appropriate, the activity relevant to this review is highlighted.

Gene Name	Species	Description
KNOTTED 1 (KN1)	Maize	KNOX Transcription Factor, meristem identity
NO APICAL MERISTEM 1 and 2 (NAM 1,2), CUC3	Maize	NAC domain, transcription factor, expressed in boundary domains
TASSELS REPLACE UPPER EARS 1 (TRU1) and TRU1-like	Maize	Ankyrin repeat domain protein expressed in the sheath and in axillary meristems.
PINFORMED 1a and 1b (PIN1a, PIN1b)	Maize	Auxin transporter
SISTER OF PIN1 (SoPIN1)	Maize	Auxin transporter
RAMOSA 2 (RA2)	Maize	Lateral organ boundary domain transcription factor, involved in axillary meristem development.
SPARSE INFLORESENCE 1 (SPI1)	Maize	YUCCA gene, auxin biosynthesis.
NARROWSHEATH 1 and 2	Maize	WOX genes, involved in leaf development
LIGULELESS1 (LG1)	Maize	Squamosa Binding Protein transcription factor, involved in ligule development.
LIGULELESS2 (LG2)	Maize	BZIP/DOG domain transcription factor, involved in ligule development.
LIGULELESS NARROW (LGN)	Maize	Serine-threonine kinase, involved in ligule development.
LIGULELESS3 (LG3)	Maize	KNOX transcription factor, ectopic expression of LG3 induces ectopic blade/sheath boundaries.
LIGULELESS4 (LG4)	Maize	KNOX transcription factor, ectopic expression of LG4 induces ectopic blade/sheath boundaries.
GNARLEY4 (GN4)	Maize	KNOX transcription factor, ectopic expression of LG4 induces ectopic blade/sheath boundaries
WAVY AURICLES IN BLADE 1 (WAB1)	Maize	TCP transcription factor, ectopic expression of WAB1 induces ectopic blade/sheath boundaries
BEL1-like homeodomain 12 and 14 (BEL12/14)	Maize	BEL1-like homeodomain transcription factors, expressed in the developing ligule
BRASSINOSTEROID INSENSITIVE 1 (BRI1)	Maize	Brassinosteroid receptor, involved in auricle development and leaf angle
BRASSINOSTEROID-DEFICIENT DWARF1 (BRD1)	Maize	Brassinosteroid C6-oxidase, involved in brassino-steroid synthesis, expressed in the base of leaves. Involved in ligule and auricle development.
BETA-D-GULCOSIDASE 1 (GLU1)	Maize	Expressed in developing ligules
UNICULME4 (CUL4)	Barley	Ankyrin repeat domain protein, expressed in the sheath and involved in ligule development
ELIGULUM A (ELIA)	Barley	RNase H domain protein, involved in ligule development
